# Corticosteroid Treatment Impact on Spinal Deformity in Duchenne Muscular Dystrophy

**DOI:** 10.1155/2014/965235

**Published:** 2014-10-29

**Authors:** Ilaria Sanzarello, Luciano Merlini, Francesco Traina, Michele Attilio Rosa, Cesare Faldini

**Affiliations:** ^1^Section of Orthopedics and Traumatology, University of Messina, 98125 Messina, Italy; ^2^Laboratory of Musculoskeletal Cell Biology, Rizzoli Orthopedic Institute, IRCCS, 40136 Bologna, Italy; ^3^General Orthopedic Surgery, Rizzoli-Sicilia Department, Rizzoli Orthopedic Institute, Bagheria, 90011 Palermo, Italy

## Abstract

Duchenne muscular dystrophy is a progressive disease with loss of ambulation at around 9-10 years of age, followed, if untreated, by development of scoliosis, respiratory insufficiency, and death in the second decade of life. This review highlights the natural history of the disease, in particular, with regard to the development of the spinal deformity and how this complication has been modified by surgical interventions and overall by corticosteroid treatment. The beneficial effect of corticosteroids may have also an impact on the clinical trial design of the new emerging causative therapies.

## 1. Introduction

Duchenne muscular dystrophy (DMD) is a fatal neuromuscular disease, affecting 1 : 3500 live male births [1]. It occurs as a result of mutations, mainly deletions, in the* dystrophin* gene. Mutations lead to absence of or defect in the protein dystrophin from the muscle fibers causing cycles of muscle fiber degeneration and regeneration with replacement by fat and connective tissue. Diagnosis is based on clinical examination observing the child run, jump, climb stairs, and get up from the floor; blood test: serum creatine kinase (CK) levels up to 50–100-fold above normal; genetic testing: approximately 65% of patients with DMD have intragenic out-of-frame (gross rearrangements) deletions and approximately 10% have duplications of one or more exons of the dystrophin gene [2, 3]; and muscle biopsy: dystrophin analysis will always be abnormal and offers a further route to confirm the diagnosis.

At the moment, there is no curative treatment for this devastating disease, and the main goal of interventions is to maintain ambulation as long as possible and to minimize the impact of the predictable complications of the disease, such as joint contractures, scoliosis, cardiomyopathy, and respiratory insufficiency.

The objective of this review is to trace the natural history of the disease, in particular, with regard to the development of spinal deformity and how this complication has been modified by surgical interventions and overall by corticosteroid treatment.

## 2. Natural History

Clinical evolution of muscular weakness in patients with Duchenne muscular dystrophy is peculiarly marked by its progressive nature. As DMD boys appear “healthy” at birth, the natural history of untreated DMD leads to the development of an abnormal gait, calf hypertrophy, and difficulty rising from the floor when at 2–5 years of age [4]. If not correctly diagnosed and treated, the boys become progressively unsteady in their walking, have a propensity to fall, use Gower's manoeuvre to stand up again, and acquire a waddling gait. Gower's manoeuvre is always present, with boys needing to turn onto their front and rise to standing from the floor using a broad-based stance, usually with the support of their hands on their thighs. Common features of the disease are calves muscle hypertrophy and, frequently, developmental delay with delayed speech. Around 9-10 years of age, the wheelchair dependence occurs [5]. Respiratory failure is the major cause of death and occurs in the second or third decade of life; it is caused by progressive respiratory muscle weakness and includes progressive restrictive ventilatory defects, chronic hypoventilation, and pulmonary infections. The remaining 10% of deaths occur due to myocardial disease and its sequelae including heart failure and dysrhythmia.

Interventions designed to lessen the predictable complications of the disease have successfully changed its course that is now compatible with survival into adult life [6]. The provision of noninvasive mechanical ventilation, assisted coughing, and cardioprotective medication allows survival into the late twenties and thirties [7]. The natural history of the disease has also been significantly changed by the use of corticosteroids (CS). The use of CS was first proposed in 1974 [8]. Efficacy has been established in improving muscle strength and timed functional tests over period of 6–18 months [9, 10]. Follow-up studies show long-term benefit with marked reduction in spinal deformity [11] and prolonged ambulation [12]. More recently, it was shown that the early use of CS has significant advantages: boys starting treatment between ages 2 and 4 maintain ambulation beyond age 16 [13, 14].

The clinical and laboratory diagnosis of DMD is now feasible much earlier than in the past and CS treatment can begin earlier in the course of the disease hopefully providing greater benefit than if treatment is delayed [15]. It should be noted in fact that the marked elevation of CK, a recognized marker of muscle fiber necrosis, is already present at birth [16, 17]. A florid dystrophic process is already evident in the muscle biopsy of newborns with DMD [16, 18]. DMD infants and young boys in the first 3 years of age have already measurable deficits in gross and fine motor function [19]. In addition, motor function declines within the first 3 years of life compared to age-matched peers [20].

## 3. Spinal Deformity

**Figure 1:**
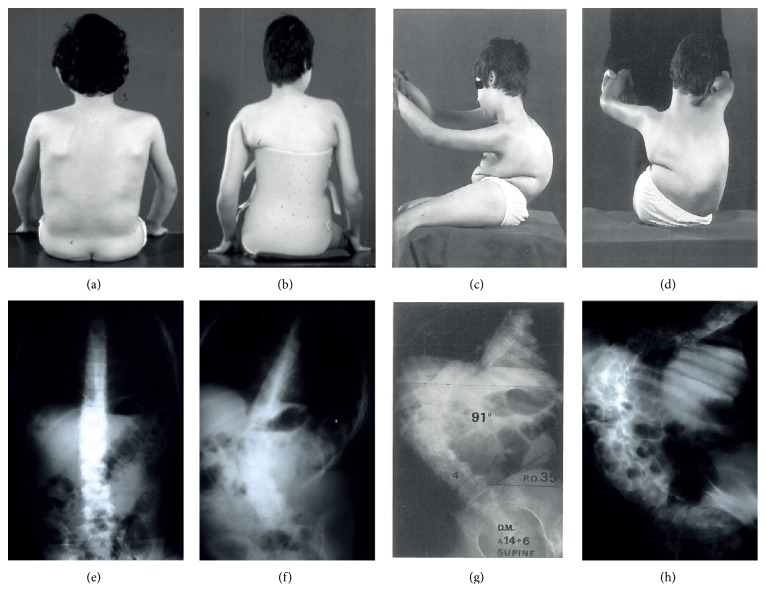
Duchenne muscular dystrophy: natural history of a progressive disease with a progressive scoliosis. A boy at 10 (a), 12 (b), and 16 years of age ((c), (d)). This boy, born in the mid-sixties, presented at 5 years of age with a 2-year story of progressive difficulty in climbing stairs and frequent falls. He was never able to run. CK was markedly elevated (more than 100 times normal). Muscle biopsy showed marked necrosis and proliferation of connective tissue. Loss of ambulation occurred at the age of 8 years and 6 months. At 10 years ((a), (e)), he had a minimal spinal curvature of 10°. He was immediately fitted with a spinal brace, which he wore during daytime. In spite of this treatment, the curvature progressed to 58° at age 12 ((b), (f)). At age 14 years and 6 months, the scoliosis measured 91°. At age 16, he was no more able to sit unsupported because of the severe collapsing spinal “C” curve of 116° with marked pelvic tilt. At age 17 years, he had pneumonia and needed ventilatory support and was subsequently left with a permanent tracheostomy. He died at age 19 years. Family history was positive for an X-linked muscular dystrophy. The mother showed big calf and persistent mild elevation of CK. A maternal uncle had a progressive muscular dystrophy and died at 15 years.

A progressive scoliosis develops in over 90% of patients as a combined result of wheel chair dependence, paralysis of the extensor muscles [21], contractures, and growth spurt (Figure 1). A severe collapsing scoliosis can interfere with breathing by reducing the lung function and can obstacle seating position, greatly decreasing quality of life of DMD patients. The spinal deformity differs from that seen in patients with idiopathic scoliosis and is described as a “C-type” curve (a gentle, sweeping curve, with apex at the thoracolumbar junction). It can cover up to 15 segments and, as a consequence, on clinical observation, be missed by the untrained eye. The spinal deformity results from a varying combination of curves in the coronal (scoliosis) and sagittal (kyphosis, lordosis) planes and excessive flexibility or stiffness of the spine due to the paralysis (collapse) or fibrous replacement (rigid spine) of the axial musculature [22]. Onset of scoliosis and loss of autonomous ambulation occur together in boys with DMD, generally between the ages of 10 and 14 years. There are different patterns of progression of scoliosis [22, 23]. In the coronal plane, scoliosis can evolve in a linear and constant way when the child is wheelchair bound or alternate a period of slow progression to a rapid increase of the curve or have a rapid evolution over a few months too. In most of the cases, the final result is a kyphoscoliosis with collapsing spine or, less frequently, a hyperlordosis or a lordoscoliosis with rigid spine [22, 24]. In addition to skeletal deformities on the sagittal and anterior-posterior plane, almost 100% of patients have a more or less distinct pelvic tilt (0°–15°). The majority of patients complain about pain when sitting in a wheelchair because of the one-sided load on one buttock. Some of them develop pressure sore formation. When scoliosis is suspected, an X-ray of the entire spine (AP and lateral view) is mandatory.

### 3.1. Prevention of Spinal Deformity

The various modalities of physiotherapy [6], including pool exercises, daily mobilization of contractures, and orthoses, like night splints or supportive sitting devices, have not shown a significant effect on progression of scoliosis. Rehabilitation in lightweight knee-ankle-foot orthoses at the point of loss of ambulation, with or without tendon release, has been proven effective in preventing/reducing progression of scoliosis during the pubertal growth spurt [25–28].

### 3.2. Effect of Nonoperative Treatment

Nonsurgical methods of spinal correction include the use of body jackets, custom-made seating inserts, and wheelchair modifications [29, 30]. Bracing is known to be ineffective to stop the progression of scoliosis in these children, as progression occurred in 94% despite bracing, and can be predicted to be 10° per year [31]. Use of bracing should be therefore reserved for patients who refuse surgery or patients who are inoperable [32].

### 3.3. Spine Surgery

Spine surgery with posterior spinal fusion [31] is the gold standard treatment for severe progressive scoliosis in DMD patients with the subsequent indications: documented curve progression, loss of seating balance, pain and/or discomfort. Surgical treatment is mainly performed to restore the balance of the spinal column in both coronal and sagittal planes, to improve life quality of patients, facilitate nursing, and improve sitting balance and comfort [24].

Instrumentation techniques have evolved over the years to achieve these goals by decreasing surgical time and blood loss with minimum neurovascular complications. Several instrumentation techniques have been applied for scoliosis correction in DMD patients ranging from halo casts with traction wires and buttons [33], Harrington rods [34], and Luque's segmental spinal fixation [35] to more recent techniques using pedicle screws and hooks [36]. Use of Harrington instrumentation technique showed significant improvement in curve correction (60% on average), delay of curve progression, and shortening of constrained postoperative recumbence period. Long-term studies have shown that this is a successful procedure with limited complications [24, 31, 37]. The most commonly described method for fusion to the pelvis is Luque rod instrumentation with use of the Galveston technique [38]. The use of the Galveston fixation with the placement of the pelvic portion of the rods between the tables of the ilia above the sciatic notch allows for correction of pelvic obliquity. Long-term studies have shown that the Luque-Galveston system with spinal fusion is an efficient, safe, well-designed, easily adaptable, and reproducible technical method for the DMD patient with a moderate spinal curve needing correction. Success rates are highest if surgery is performed early, when the spine is still mobile at a Cobb angle of 20–40° and the cardiac and respiratory function is, in part, preserved [31]. The rate of complications is so reduced and if there is a pelvic obliquity, the fixation to the pelvis is always indicated. The search for a safe and resistant surgical technique has led to the advent of pedicle screws and hook system [39]. Pedicle screws are penetrating anchors, which are superior to gripping fixation obtained by laminar wires and cables. This system ensures greater resistance and a good biomechanical stability [40]. The pedicle screw system was still found to be superior in achieving a better major curve correction and lesser neurological complications [41]. Overall, the described instrumentations appear to provide and maintain an optimal degree of correction at medium to long-term follow-up but the advantages of lowest blood loss and least surgical time without the need for pelvic fixation seem to swing the verdict in favor of the pedicle screw system [36]. Patients with DMD have increased blood loss during spinal surgery compared to non-DMD patients. In Duchenne patients, Labarque et al. [42] found that platelets have a disorganized cytoskeleton due to dysfunctional dystrophin that may result in increased bleeding during surgery. Independently of the surgical technique, patients operated with a scoliotic curve less than 40° had better results, even in time, than patients operated with a more severe scoliotic curve (40° or more) [31].

### 3.4. Respiratory Function and Spine Surgery

**Figure 2:**
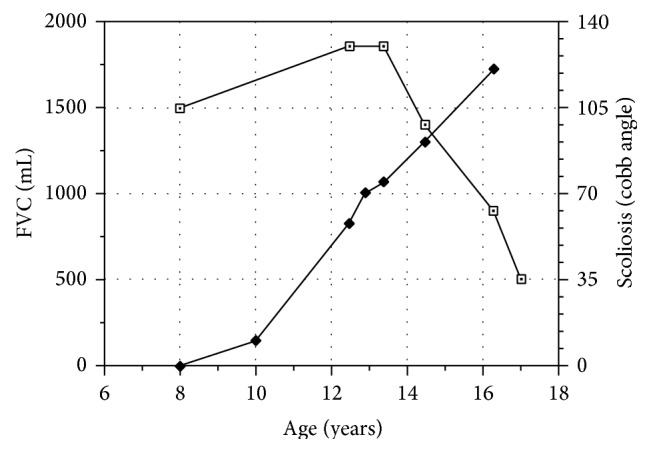
Duchenne muscular dystrophy: progressive course of scoliosis and respiratory compromise. Data of the same DMD boy of Figure 1. His forced vital capacity (FVC) increased up to the age of 12 years then rapidly declined. A scoliotic curve started at the age of 10° years and continued to worsen in spite of spinal bracing.

Patients with DMD develop a restrictive respiratory pattern with reduction of maximal respiratory pressures and forced vital capacity (FVC) that eventually causes respiratory insufficiency and death (Figure 2). In these patients, lung function increases up to the age of 10–12 years hitting its plateau; then lung function decreases with an estimated loss of 10% per year of FVC [43]. No correlation has been found between grade and progression of respiratory dysfunction and severity of scoliosis, because intrinsic respiratory muscle's weakness is actually the main determinant of decline in respiratory function in DMD. Kennedy et al. [44], in a study on spinal surgery and lung function, reported no differences between surgical group and nonsurgical group in the rate of deterioration of %FVC which was 3–5% per year concluding that spinal stabilization in DMD does not alter the decline in pulmonary function nor does it improve survival. In addition, no difference was found in the rate of vital capacity decrease between operated or nonoperated patients and, in operated patients' subgroup, between patients with scoliotic curve less than 40° and patients with more severe scoliotic patterns (>40°) [24].

In spite of these progresses, spinal stabilization in patients with DMD remains a demanding surgery because of dystrophic involvement of the paraspinal muscles and vascular smooth fibers, abnormal platelet function with a higher risk of intraoperatory bleeding [42] requiring an adequate surgical technique, and a proper availability of blood units [45]. Intraoperative bleeding is the main risk factor; however, several other complications are common in spinal surgery in DMD patients: intraoperative infection, implant failure, vertebral fracture, pullout of the screws, sacral decubitus, and death [46, 47]. This surgery should be performed only by experienced surgeons and highly specialized centers; moreover, it does not influence the natural course of respiratory decline.

## 4. Corticosteroids in DMD

The first scientific evidence on the beneficial effect of steroids in Duchenne muscular dystrophy was documented over 40 years ago by Drachman et al. [8]; since then, several other studies have demonstrated the efficacy of corticosteroid therapy in delaying the loss of independence and autonomous ambulation and in maintaining an adequate pulmonary function [15]. Prednisone or deflazacort has been demonstrated to have a beneficial effect on muscle strength and function in boys with DMD and should be offered as treatment [15, 48]. Currently, corticosteroids are the gold standard treatment for muscle weakness in ambulant children with DMD. The most common daily dosage regimes are 0.75 mg/kg/day prednisone/prednisolone and 0.9 mg/kg/day deflazacort [15]. Other studies, using steroids with various combinations of daily, alternate-day, or cyclical prednisone treatment [13, 49–51], have also demonstrated benefit in functional parameters. However, despite the fact that corticosteroids are routinely prescribed to DMD patients in most countries, there is no consensus on the optimal age to initiate treatment, optimal dose, and optimal dose schedule [6, 15].

Although most of the different schedules/dosages claim to be effective at improving muscle strength and function, none has been shown to be able to maintain this result with time. All long-term studies, independently of schedule/dosage, have shown that after a variable period of “improvement,” patients invariably lose muscle strength and function, although at a lower rate compared to patients that had not taken corticosteroids. The effect of corticosteroid treatment, at its best, is only able to slow the progressive course of the disease. This has been documented in a long-term study of alternate-day corticosteroids in five 2- to 4-year-old DMD patients [13, 14, 52]. The primary outcome measure of the study was prolongation of the ability to walk. One patient lost ambulation at age 10. Four patients, aged 16 to 18 were fully ambulant, and 3 of them could still climb stairs. Short stature and delayed puberty were the most relevant side effects [14]. This and other studies [15, 48] suggest that long-term corticosteroid treatment is effective in prolonging function but not in recovering lost function and, therefore, its early use seems appropriate.

### 4.1. Mechanism of Action of Corticosteroids

The exact mechanisms by which steroids slow the dystrophic process are still under investigation [53]. Various possibilities have been proposed based mainly on observations in mouse models of muscular dystrophy and a limited number of studies in patients [48]. The effects of steroids in animal models include attenuating muscle fiber necrosis [54]; decreasing the entry of calcium into cells [55, 56]; regulating gene expression [57]; reducing cytotoxic T lymphocytes [58]; increasing laminin expression and myogenic repair [59]; decreasing muscle apoptosis and cellular infiltration [60]; protecting against mechanically induced fiber damage (possibly by stabilizing the muscle fiber membranes) [61]; increasing muscle levels of taurine and creatine [62]; reducing muscle degeneration and increasing survival [63]; alleviating myofiber pathology by activation of the calcineurin/NF-AT pathway [64]; increasing the number of myoblasts [65]; enhancing the myogenesis of satellite cells; and increasing dystrophin-related protein expression [66]. In DMD muscle/cell, the effects of steroids include postrascriptionally mediated utrophin accumulation [67]; increasing muscle mass by inhibition of muscle proteolysis [68, 69]; enhancing dystrophin expression [70]; inhibiting myotube death during myogenesis [71]; and reducing the number of mononuclear inflammatory cells and dendritic cells [72]. It is unlikely that the effect of prednisone results from its immunosuppressive action given that azathioprine decreases mononuclear subsets infiltrating muscle to a similar degree as does prednisone, although azathioprine-treated patients do not show a clinical improvement [73].

### 4.2. Side Effects of Corticosteroids

Weight gain is the most frequently reported side effect for DMD children on steroids. It is important to support parents with adequate dietary counseling to limit weight gain, to cut down on high calorie foods, and to maintain a healthy diet. Long-term daily use of steroids has an effect on a loss of final height at the end of growth. In several comparative studies, children who had used steroids were shorter by >10 cm compared to children who had not undertaken this therapy. Bone mineral density is one of the most important side effects related to the steroids; however, it was also demonstrated that the bone density reduction occurs in DMD even before steroid use [74]. This is probably caused by the low muscle activity present in these patients. Cataract is another complication suffered by children with DMD, and, for this reason, ophthalmological examination is necessary to monitor for the development of cataracts and increased intraocular pressure. Other common side effects include cushingoid appearance, hirsutism, acne, behavioral changes consisting of irritability and hyperactivity, osteonecrosis, and hypertension.

### 4.3. Corticosteroids' Effect on Spinal Deformity

The positive effect of corticosteroid treatment in the prevention/delay of the development of scoliosis deformity has been recognized by all long-term studies [11, 75–78]. Of particular interest is the Canadian deflazacort study involving 54 DMD boys who were followed for 15 years [12, 75, 79–81]. Fifty-four DMD boys aged 7 to 10 and able to walk were enrolled in a nonrandomized comparative study; thirty patients were treated with deflazacort (treatment group), and twenty-four were not (control group). Patients in the treatment group had a better pulmonary function; they were able to walk longer and to climb stairs for a mean of 1.5 years longer compared to patients in the control group. At the last follow-up (fifteen years), six (20%) in the deflazacort group and twenty-two (92%) in the control group developed scoliosis and underwent spinal surgery. Kinali et al. reported a lower prevalence and an average milder scoliotic curve in patients treated with steroids [82]. Houde et al. [78], in another study of deflazacort use, report that scoliosis was much less severe in treated (14 ± 2.5°) than in untreated boys (46 ± 24°). King et al. [11], examining the orthopedic outcomes of long-term daily corticosteroid treatment in DMD, showed that treated boys had a significantly lower prevalence of scoliosis than the untreated group (31 versus 91%). The average scoliotic curve was also significantly milder in the treated group (11.6°) compared with the untreated group (33.2°) [11]. Moreover, in another recent cohort study that analyzed the effect of prednisone or deflazacort, boys who had received steroid therapy were significantly less likely to undergo spinal surgery [77].

### 4.4. Corticosteroids Effect on Respiratory Function

Steroid therapy seems to be effective in preserving respiratory muscle strength in DMD, even if it remains uncertain how long this effect can be sustained over time. Respiratory outcome studies of DMD patients treated with steroids showed improved values of % FVC. In a retrospective study including forty-nine DMD patients treated with corticosteroids for 7 years, Balaban et al. [83] showed that both deflazacort and prednisolone had a beneficial long-term effect on pulmonary function. Long-term steroid therapy is also associated with improved peak cough flow and respiratory muscle strength in patients with DMD [84]. A recent similar study also showed that CS can stabilize or delay the loss of lung function even in nonambulant patients or patients older than 10 years and in those treated after 7 years of age [85].

## 5. New Emerging Therapies

The most promising therapies for DMD are gene therapy, exon skipping, and stop codon read-through, all aiming at restoring the expression of dystrophin. A drisapersen phase III clinical trial (NCT01254019), with 186 patients, aiming to induce skipping of exon 51 and* de novo* dystrophin production in patient muscle, failed to show significant improvement of the primary outcome measure, the 6-minute-walk test [86]. Eteplirsen, targeting skipping of exon 51, showed variable levels of dystrophin restoration and stabilization of clinical outcome in a subset of patients in an open-label extension study [87]. However, it remains to be seen whether eteplirsen can maintain a significant clinical benefit with time. The ataluren trial (stop codon read-through) with 174 patients showed a marginally significant improvement in the 6-minute-walk test compared to placebo. However, this drug has shown very little evidence of dystrophin restoration and the trial utilized a very subjective scoring method [87, 88].

The failure of the only phase III study of antisense oligonucleotide (drisapersen) performed so far has raised much discussion about the validity of dystrophin as a biomarker and the 6-minute-walk test as an outcome measure [86–88]. It has also been suggested to optimize aspects of the clinical trial design, including younger age of treatment, because older boys have fewer myofibers left to rescue [86].

However, two critical points have yet to be considered. First, the inclusion of patients on CS, both in the treatment and in the control groups, may be problematic. This setting supposes that if the active treatment has a positive effect, it will be added to the already known positive effect of CS. If this is not the case, we can miss the opportunity to use an effective treatment possibly with fewer side effects compared to CS. Second, the choice of the clinical outcome measures should match the type of improvement that is expected. In a rapidly progressive disease like DMD, treatments should be considered effective if they are able to slow progression. Muscle strength (maximal isometric muscle force) and muscle function (6-minute-walk test) are not appropriate endpoints for any intervention that has an impact limited to slow progression. Muscle strength and muscle function, in this situation, may have a transient improvement at some point in time; however, they are expected to deteriorate with time, although at a lower rate compared to no treatment. Some endpoints suitable to demonstrate slowing progression of the disease are (1) survival to death; (2) survival to death or any respiratory intervention; and (3) prolongation of independent walking. It is evident that all these endpoints require a long period of treatment particularly if treatment is started early in the course of the disease.

## 6. Conclusions and Future Direction

In DMD patients, the development of spinal deformity has been dramatically changed by the progressive diffusion of CS treatment. There is now a general consensus that long-term CS therapy (1) prolongs ambulation, (2) reduces the need for spinal surgery, (3) reduces cardiopulmonary dysfunction, (4) delays the need for mechanical ventilation, and (5) increases survival and quality of life of DMD patients. Recent findings also indicate that early use of CS has significant advantages.

The goal of future DMD treatments should be to find a product at least as good as glucocorticoids with a lower side effect profile or with a significant glucocorticoid sparing effect. Along this road, the new emerging and promising treatments are nonsense suppression therapies for boys with premature stop codon mutations and exon skipping by means of antisense oligonucleotides. There are plenty of lessons to be learnt from the recent failure of a phase III exon skipping clinical trial, which should help to overcome the roadblock. First, there is the urgent need to standardize methods for dystrophin quantification and optimize several aspects of the clinical trial design. Second, approvals of exon skipping and splice modulation as therapies for DMD require that a correlation be shown between dystrophin expression and clinical outcomes. But while restoration of dystrophin can be verified quickly, prolongation of walking, that is, the desirable clinical outcome, will take 10 or more years to be shown if treatments are started early. Because of this unavoidable misalignment in time, accelerated approval should be based on surrogate biochemical evidence only (*de novo* dystrophin demonstration in muscle). In addition placebo controlled trials will be unfeasible if a decade or more of blindness is needed to show slowing of disease progression in the treated group compared to placebo.
